# An M2e-based synthetic peptide vaccine for influenza A virus confers heterosubtypic protection from lethal virus challenge

**DOI:** 10.1186/1743-422X-10-227

**Published:** 2013-07-09

**Authors:** Ji-Hong Ma, Fu-Ru Yang, Hai Yu, Yan-Jun Zhou, Guo-Xin Li, Meng Huang, Feng Wen, Guangzhi Tong

**Affiliations:** 1 Division of Swine Infectious Diseases, Shanghai Veterinary Research Institute, Chinese Academy of Agricultural Sciences, Shanghai 200241, China

**Keywords:** Influenza A virus, Influenza, M2e, Synthetic peptide vaccine

## Abstract

**Background:**

Vaccination is considered as the most effective preventive method to control influenza. The hallmark of influenza virus is the remarkable variability of its major surface glycoproteins, HA and NA, which allows the virus to evade existing anti-influenza immunity in the target population. So it is necessary to develop a novel vaccine to control animal influenza virus. Also we know that the ectodomain of influenza matrix protein 2 (M2e) is highly conserved in animal influenza A viruses, so a vaccine based on the M2e could avoid several drawbacks of the traditional vaccines. In this study we designed a novel tetra-branched multiple antigenic peptide (MAP) based vaccine, which was constructed by fusing four copies of M2e to one copy of foreign T helper (Th) cell epitope, and then investigated its immune responses.

**Results:**

Our results show that the M2e-MAP induced strong M2e-specific IgG antibody,which responses following 2 doses immunization in the presence of Freunds’ adjuvant. M2e-MAP vaccination limited viral replication substantially. Also it could attenuate histopathological damage in the lungs of challenged mice and counteracted weight loss. M2e-MAP-based vaccine protected immunized mice against the lethal challenge with PR8 virus.

**Conclusions:**

Based on these findings, M2e-MAP-based vaccine seemed to provide useful information for the research of M2e-based influenza vaccine. Also it show huge potential to study vaccines for other similarly viruses.

## Background

Influenza virus is a globally important respiratory pathogen which causes a high degree of morbidity and mortality in humans and animals annually [1]. Influenza virus typically infects 10~20% of the total worldwide population during seasonal epidemics, resulting in three to five million cases of severe illness and 250,000 to 500,000 deaths per year [2]. Moreover, novel influenza strains appear occasionally in the human population, causing pandemics. In 2009, the world confronted the first influenza pandemic of the 21st century, which iscaused by a novel influenza A H1N1 virus [3]. Antigenic and genetic analysis has suggested that this pandemic H1N1 virus is a product of reassortment between genes of the human, avian and swine influenza strains [4].

To date, vaccination is considered as the most effective preventive measure to control influenza. However, conventional vaccines have many drawbacks, the most important is the uncertainty of virus selection strains to be included in each year’s vaccine formulation [5]. The hallmark of influenza virus is the remarkable variability of its major surface glycoproteins, HA and NA, which allows the virus to evade existing anti-influenza immunity in the target population [6]. The potential shortage of pandemic influenza vaccines and the absence of specific-immunity in the human population make the development of a cross-protective influenza vaccine, which is based on conserved antigens, a promising prophylactic strategy. M2e, the ectodomain of the M2 protein, is highly conserved across influenza a subtypes and has become an attractive antigen target for producing a cross-protective influenza vaccine conferring broad spectrum prevention [7-9]. In contrast to BALB/c mice, Wolf AI, et al. show that immunization of other inbred and outbred mouse strains did not induce protective Abs. So it suggested that it correlated with a defect in T cell but not B cell responsiveness to the M2e-MAPs [10].

In this study we designed a novel tetra-branched multiple antigenic peptide (MAP) based vaccine, which was constructed by fusing four copies of M2e to one copy of foreign T helper (Th) cell epitope. The vaccine can provide heterosubtypic protection against lethal virus challenge.

## Results

### M2e-MAP immunization could induce high titers of M2e-specific IgG antibodies

**Figure 1:**
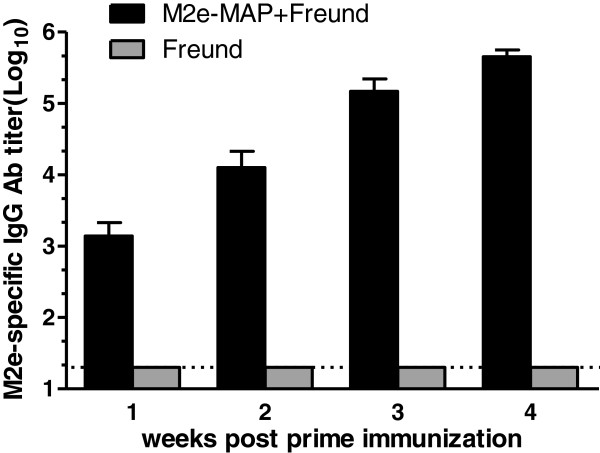
**M2e-specific antibody titers induced by M2e-MAP vaccine.** Mice were vaccinated with M2e-MAP plus Freund adjuvant (s. c.). Mice receiving Freund were used as negative controls. Sera were collected at 1, 2, 3, 4 weeks post first immunization. The titers were expressed as the highest serum dilution which is greater than twice the average absorbance value at OD450 nm of pre-vaccination sera. The data are expressed as geometric mean titer (GMT) ± standard deviation (SD) of 10 mice per group. The lower limit detection (1:20) is indicated by a dotted line. Experiments were repeated three times.

To evaluate humoral immune responses induced by M2e-MAP, mice were vaccinated with 10 μg of M2e-MAP plus Freund’s adjuvant as described in Methods, and M2e-specific IgG antibodies were detected in mouse serum samples by ELISA. As shown in Figure 1, M2e-MAP induced strong M2e-specific IgG antibody responses, with the titer of 1:10^3^ 7 days post first immunization, then the titer reached of 1:10^4^ before boost, and increased to the highest titer over 1:10^5^ 14 days post the boost immunization. In contrast, only background level of antibody responses was detected in the mice vaccine with Freund’s adjuvant alone.

### M2e-MAP vaccination limited replication of virus and attenuated virus-induced lung pathology

**Figure 2:**
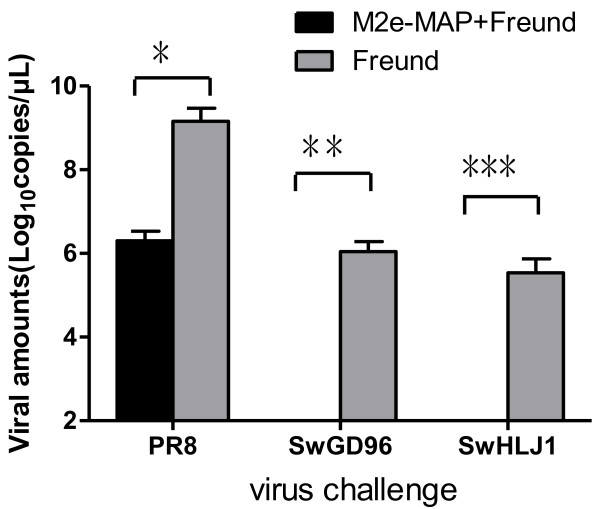
**Viral amounts in lungs on day 3 post challenge.** 5 mice of every challenge group were euthanized 3 days post-challenge and the viral amounts of lungs were determined by real time PCR. The values were expressed as the mean log10 viral copies/μL ±S.D of 5 mice per challenge group. *means in PR8 challenged group P < 0.001 compared to the Freund’s adjuvant control; **means in SwGD96 challenged group P < 0.001 compared to the Freund adjuvant control; ***indicates in SwHLJ1 challenged group P < 0.001 compared to the Freund adjuvant control.

Two weeks post boost immunization, mice were challenged with a lethal dose (10LD_50_) of PR8 and 10^6^EID_50_ of SwGD96 and SwHLJ1. The vaccinated mice were sacrificed three days after challenge and lung tissues were collected for the detection of viral titers and histopathological examination. Viral titers were determined in SPF eggs and real time PCR methods [11]. In the control group, 3 different types of virus replicated to titers from 10^2.15^ to 10^5.80^ EID_50_/ml in lungs. In contrast, in SwHLJ1 and SwGD96 challenged group, virus cannot detect in lungs of mice immunized with M2e-MAP (<1.0 log10 EID_50_/ml) and the titer in lungs dropped to 10^3.60^ EID_50_/ml in PR8 challenged mice(Table 1).Compared with the adjuvant control group, the average viral amounts in lungs of M2e-MAP-vaccinated mice was significantly lower (P < 0.001) after challenge with SwHLJ1, SwGD96 and PR8 viruses (Figure 2), which suggested that the M2e-MAP can induce potent protective immunity against viral replication following infection with different types of influenza A virus.

**Table 1:** Virus isolation and titrations in lungs on day 3 post challenge

**Immunogen**^**a**^	**Virus isolation/total**			**Protection against challenge and virus titer**^**b**^		
				**(log10 EID50/ml±S.D.)**		
	**PR8**	**SwGD96**	**SwHLJ1**	**PR8**	**SwGD96**	**SwHLJ1**
M2e-MAP+Freund	5/5	0/5	0/5	3.60±0.22	<1	<1
Freund	5/5	5/5	5/5	5.80±0.27	3.26±0.29	2.15±0.34

a. 60 mice per group were injected twice subcutaneously with M2e-MAP plus Freund and Freund as control.

b. Mice were challenged intranasally 2 weeks post 1st boost with 106EID50 of SwGD96, SwHLJ1 and 10LD50 of PR8 viruses. Virus titer of lungs was determined 3 days later in ECE. The values were calculated by the method of Reed-Muench and expressed as the mean log10 EID50/ml±S.D. The lower limit of detection of virus was 1.0 log10 EID50/ml.

**Figure 3:**
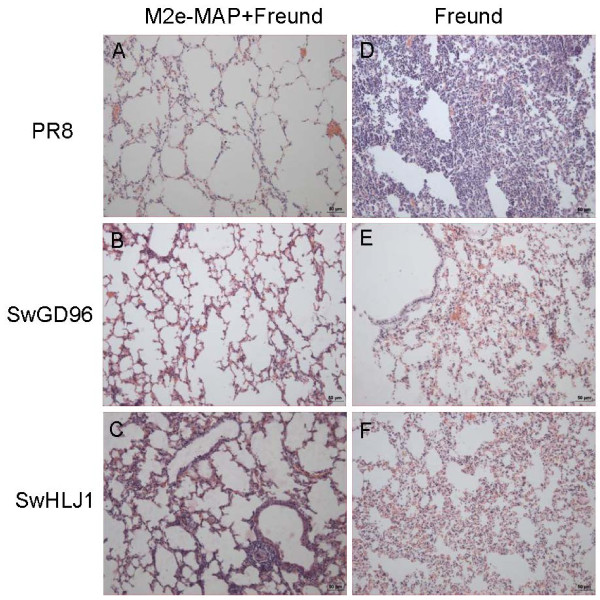
**Histopathological changes in the lungs of virus challenged mice.** Immunized mice were challenged by PR8 (**A** and **D**), SwGD96 (**B** and **E**) and SwHLJ1(**C** and **F**) and lungs were collected for histopathological analysis 3 days post challenge (HE stain; bar = 50 um).The figure indicates the representative imagines of histopathological observations of M2e-MAP plus Freund immunized mice or Freund only.

Histopathologic examination showed that PR8 challenged mice, adjuvant control group revealed dramatic histopathological damages including large inflammatory cell infiltration, lung tissue necrosis, abscess formation and accompanied by bleeding, and some lungs tissue showed compensatory emphysema (Figure 3D). In contrast, the M2e-MAP-vaccinated mice lungs exhibited relatively minor pathological changes, with only slight expansion of alveoli, and the interval of alveolar became slightly flat because of pressure (Figure 3A). The vaccine also protected mice against infection by SwHLJ1 and SwGD96 virus, the M2e-MAP-vaccinated mice showed no obvious pathological damage (Figure 3B and C). On the contrary, the adjuvant control mice appeared interstitial pneumonia, alveolar capillaries and small blood vessel wall dilatation and congestion, accompanied by a small amount of bleeding (Figure 3E and F). The above data suggested that M2e-MAP vaccination may protect the mice against challenge of SwHLJ1, SwGD96 and PR8 virus through a combination of limiting viral replication in the lungs and attenuating virus-induced lung pathology.

### M2e-MAP vaccination provided effective protection from lethal challenge with PR8 virus

**Figure 4:**
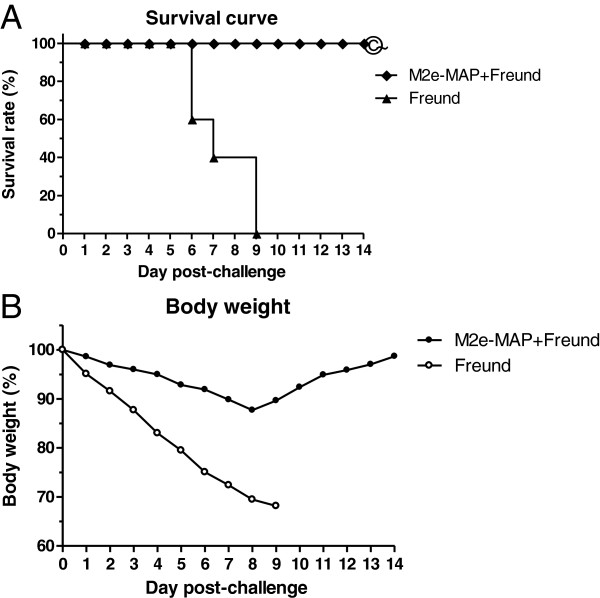
**Survival curve and body weight in PR8 challenged mice.** Survival curve and body weight in PR8 challenged mice. Mice were challenged with 10LD50 of PR8 virus intranasally and monitored daily for 2 weeks post challenge. **A**. Survival rate. The significant different (P<0.0001) of M2e-MAP+Freund versus Freund alone is indicated as *. **B**. Percentage (%) of mouse body weight. Each point represents mean of 10 mice per group.

To further confirm the protective immunity of M2e-MAP vaccination against lethal infection, mice received lethal challenge (10LD_50_) of A/Puerto Rico/8/34(PR8; H1N1) strain were monitored for weight loss and death for the subsequent two weeks postchallenge. As shown in Figure 4B, mice vaccinated with M2e-MAP in Freund’s adjuvant rallied from body weight loss eight days after virus infection. In contrast, body weight of the mice in the adjuvant control group dramatically decreased, almost 30%, in some cases. All mice received adjuvant alone died within 9 days after lethal virus challenge. In comparison, 100% of the mice survived in the groups vaccinated with M2e-MAP, with the survival rate significantly different from the matched adjuvant control (P < 0.001) (Figure 4A). These data illustrated that M2e-MAP could afford protection against lethal challenge of PR8 virus.

## Discussion

The considerable antigenic variation in influenza A virus and other virus such as FMDV and HIV presents problems in their control by vaccination [12]. In previous researches anti-M2e Abs induced by M2e-MAPs shows highly cross-reactive and can mediate protection to various viruses [10]. In the case of influenza A virus, an attractive alternative approach would be to develop a cross-protective influenza vaccine based on conserved antigens such as M2e. Several studies have shown that immunization with M2e can protect against influenza A virus infection [13]. Different results and conclusions from previous studies may be due to the difference of carrier proteins, adjuvant or routes of administration. The vaccine described in the present study has several advantages. The researchers showed that branched MAP based vaccine is better than single peptide based vaccine, and results also demonstrated that MAP-based vaccine could give high-titer antibody responses [8]. And vaccines with T-helper epitope can induce an antibody response [14]. So we have designed the vaccine by fusing four copies of M2e to one copy of foreign T helper (Th) cell epitope.

In this study, virus titers were determined by two methods, the first one is the traditional way by SPF eggs, and the second method was real time PCR which was showed previously [11]. Bothexperiments showed that the M2e-MAP could induce strong M2e-specific IgG antibody, which responses following 2 doses immunization in the presence of Freund and limited viral replication. Also it could attenuate histopathological damage in the challenged mice lungs and was able to counteract weight loss and protect mice from lethal challenge with PR8 virus. Therefore, the induction of M2e-specific antibody responses was necessary for M2e-based vaccines in the prevention of these three virus infection.

Several distinct M2-based vaccine constructs have been developed and found that they could induce significant resistance to influenza type A virus replication in mice [15-19]. We routinely used a fairly severe viral challenge to evaluate the efficacy of the M2e-based vaccination. It may be noted that the challenge conditions used by various viruses to score protection after vaccination with an M2e-based vaccine vary widely [20-22]. In this study high titers M2e-specific antibody responses could be induced following immunization of M2e-MAP plus Freunds’ adjuvant. Using three virus strains to challenge the immuned mice and got good results. After challenged by PR8 virus, a highly lethal virus, it demonstrated a clear-cut protection against mortality. The rest two mild virulence virus strains did not allow to score for survival, but the protection was assayed by reduction of virus titer in lungs homogenates. So we can know that the M2e-MAP play important role in the development of the vaccine.

In conclusion, we designed a novel tetra-branched multiple antigenic peptide (MAP) based vaccine, which was constructed by fusing four copies of M2e to one copy of foreign T helper (Th) cell epitope. This study provided useful information for the further development of M2e-based influenza vaccine.

## Materials and methods

### Virus and peptide

**Figure 5:**
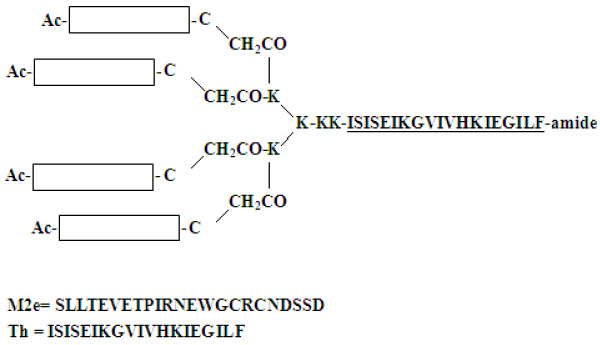
**Structure of the synthetic M2e-MAP.** The M2e-MAP was synthesized on [Fmoc-Lys (Fmoc)] 2-Lys-Cys (Acm)-βAla-Wang Resin in a tetra-branched form, which carries four copies of M2e, those four copies of M2e were fused to one copy of foreign T helper (Th) cell epitope.

A/Swine/Heilongjiang/1/05(SwHLJ1; H3N2), A/Swine/Guangdong/96/06(SwGD96; H1N1)were isolated from China [23,24]. A/Puerto Rico/8/34(PR8; H1N1) is a mouse-adapted virus. Viruses were grown in allantoic fluids of 10-day-old SPF embryonated chicken eggs at 37°C for 3 days. Virus were harvested and stored in aliquots at −80°C. The 50% lethal dose (LD_50_) of PR8 was determined in mice after serial dilutions of the virus stock and the result was calculated by Reed Muenchmethod [25]. The tetra-branched M2e-MAP was constructed by fusing four copies of a universal sequence which is an extracellular part of M2 protein with one copy of promiscuous T helper (Th) cell epitope [26] by a lysine tree with two additional Lys residues defining a putative cleavage site for cathepsin D [27]. Then it was synthesized by Genscript Co., Ltd (China). The synthesized peptide was prepared at 10 mg/mL in distilled water and stored at −20°C.The structure of M2e-MAP is shown in Figure 5.

### Immunization and virus challenge

Six to eight weeks old female SPF BALB/c mice were purchased from the Sino-British SIPPR/BK Animal Co., Ltd (Shanghai, China) and were immunized subcutaneously (s. c.) with M2e-MAP (10μg per mouse) plus Freund’s complete adjuvant (FCA, sigma) at a final volume of 100 μL and boosted with the same amount of immuogen in Freund’s incomplete adjuvant (FIA, Sigma) at a 2-week intervals. Mice injected with Freund’s adjuvant alone were used as negative control. Mice sera were collected at 0,1,2,3 and 4 weeks post first immunization to detect specific antibody responses.

Two weeks after boost, mice were anesthetized and challenged intranasally (i.n.) with a lethal dose (10LD_50_) of PR8 and 10^6^EID_50_ of SwGD96 and SwHLJ1. Challenged mice were observed for illness or death and weighed daily for 2 weeks. Lung tissues were collected from euthanized mice on day 3 post-challenge for further virological tests and histopathological analysis.

### Antibody detection

The M2e-specific antibody titers were determined by endpoint ELISA. Briefly, 96-well microtitre plates (Costar) were coated with peptide (0.5 μg per well) in 0.1 M carbonate buffer (pH 9.6) overnight at 4°C. After blocking with 5% skim milk in PBS for 2 h, serial diluted mice sera samples were added and incubated for 1 h at 37°C. Washing the ELISA plate, andhorseradish peroxidase (HRP)-labeled goat anti-rabbit IgG (1:10,000 dilution, Beijing Zhongshan Biotechnology) was added and incubated for 1 h at 37°C. Assay was developed using 3, 3′, 5, 5′-tetramethylbenzidine (TMB) (Amresco), and the reaction was stopped by adding 2 M H_2_SO4. The absorbance at 450 nm was measured by microplate autoreader (BioTek Instruments). The reciprocal of the highest dilution of the serum that is greater than twice average absorbance value of pre-vaccination serum was designated as the antibody titer.

### Virus titers in lung

Lung tissues from euthanized mice were aseptically removed and homogenized in 1 ml of cold PBS. The homogenates were frozen at −80°C and later thawed for ease of handing. Solid debris was pelleted by centrifugation. Serial diluted from initial dilutions of 1:10 and then titrated for virus infectivity in 10-day-old SPF ECE. Virus titers were calculated by the Reed-Muench method and expressed as mean log10EID_50_ per milliliter ± standard deviation (SD).

In addition, lung tissues were also collected and tested by real-time RT-PCR assay recommended by WHO with some modifications as described [11]. Lung tissues were homogenized in 300 μL of cold PBS and viral RNA were extracted by RNeasy Plus Mini kit (QIAGEN). Then reverse transcription were performed and viral amounts in lungs were determined by real-time PCR using the InfA primers and TaqMan probe. The specific primers and labeled fluorogenic-probe were as follows: InfA-F:5-GACCGATCCTGTCACCTCTGAC-3; InfA-R:5-AGGGCATTCTGGACAAAGCGTCTA-3; TaqMan probe:5-FAM-TGCAGTCCTCGCTCACTGGGCACG-BHQ-3.

### Histopathological analysis

The lung tissues of challenged mice were removed and immediately fixed in 10% neutral buffered formalin, and then embedded in paraffin wax. Sections were made at 4–6 μm thickness and mounted on slides. Histopathological changes were examined by H & E staining and observed under light microscopy.

### Statistical analysis

The survival curves were calculated by Kaplan-Meier method and the significance were analyzed with log-rank test. Other data were analyzed by two-tailed Student’s t test. P < 0.0001 was considered significantly different. All analysis were performed by Graphpad Prism software.

### Ethical approval

In this study, all of the slaughter experiments were conducted in accordance with the guidelines of Shanghai Veterinary Research Institute, Shanghai, china (permit number SYXK 2011–0116). All animal procedures were approved by the Animal committee of Shanghai Veterinary Research Institute.

## Competing interests

The authors declare that they have no competing interests.

## Authors’ contributions

GT and YH have been involved in conception and design of the study, and revising the manuscript critically; JM has been involved in design of this study, performed the study and collected data, and prepared the first draft of the manuscript; FY participated in design of the study, and completed the final manuscript. GL, MH and BW participated in data analysis. All authors read and approved the final manuscript.
